# Emerging Roles of tRNA Modifications in Translational Adaptation of Human Fungal Pathogens

**DOI:** 10.3390/microorganisms14091972

**Published:** 2026-09-07

**Authors:** Yuqi Zhu, Jiaqi Chen, Xinran Li, Ling Lu, Yuanwei Zhang

**Affiliations:** Jiangsu Key Laboratory for Pathogens and Ecosystems, Jiangsu Engineering and Technology Research Centre for Microbiology, College of Life Sciences, Nanjing Normal University, Nanjing 210023, China; 251201016@njnu.edu.cn (Y.Z.); cjq3137047897@163.com (J.C.); 261202100@njnu.edu.cn (X.L.); linglu@njnu.edu.cn (L.L.)

**Keywords:** tRNA modification, human fungal pathogens, translational control, *Aspergillus fumigatus*, *Candida albicans*, *Cryptococcus neoformans*, antifungal response, virulence

## Abstract

Invasive fungal infections can be life-threatening, particularly in patients with impaired immunity, yet treatment remains limited to a few antifungal drug classes. During infection, fungal pathogens encounter changes in temperature, nutrient availability, immune pressure and drug exposure that challenge their growth and survival. tRNA modifications may contribute to adaptation by supporting tRNA stability, aminoacylation and codon decoding. Studies in *Aspergillus fumigatus*, *Candida albicans* and *Cryptococcus neoformans* have linked conserved tRNA-modification pathways to fungal development, host interaction, stress adaptation, virulence and the response to 5-fluorocytosine, with evidence ranging from genetic and infection phenotypes to biochemical characterization of modification reactions. In this Review, we examine how these studies connect modification chemistry and substrate tRNAs with translation, protein output and infection phenotypes. We also draw on mechanistic studies from model organisms and plant-pathogenic fungi to clarify translational links that remain unresolved in human fungal pathogens. We conclude by discussing when tRNA-modification pathways become functionally limiting under host-associated conditions and how they may alter antifungal responses.

## 1. Introduction

Invasive fungal infections remain difficult to treat because few antifungal drug classes are available, resistance is increasing and mortality remains high among patients with impaired immunity [1,2,3,4,5]. *Aspergillus fumigatus*, *Candida albicans* and *Cryptococcus neoformans* are listed as critical-priority fungal pathogens by the World Health Organization [1]. Their infection strategies differ markedly. Inhaled *A. fumigatus* conidia germinate into tissue-invasive hyphae, *C. albicans* alternates between yeast and filamentous forms, and *C. neoformans* can survive within phagocytic cells before disseminating to the central nervous system [6,7,8]. Despite these differences, all three pathogens encounter changes in temperature, nutrient availability, immune pressure and antifungal exposure during infection.

Fungal adaptation to host-associated conditions is commonly studied through gene-expression and signaling networks that regulate growth, morphogenesis, metabolism and stress resistance [9]. RNA modifications can act after transcription by altering RNA processing, stability and translation, with consequences for interactions between pathogens and their hosts [10]. tRNAs are especially relevant because they are among the most extensively modified cellular RNAs. More than 100 chemically distinct modified nucleosides have been identified in tRNA, many of which contribute to folding, structural stability, aminoacylation or codon decoding [11,12,13,14,15]. These modifications are installed by methyltransferases, pseudouridine synthases, acetyltransferases, deaminases, transglycosylases and multienzyme pathways that add side chains or sulfur groups [11,12,15]. Positions 34 and 37 in the anticodon loop are functionally important modification sites because they affect wobble pairing, decoding accuracy and reading-frame maintenance. Modifications elsewhere in the molecule help preserve tRNA structure and can influence aminoacylation or delivery of charged tRNAs to the ribosome [11,12]. A defect in a modifying pathway can therefore reduce the amount of a mature tRNA, impair its use in translation or alter decoding of the codons it normally reads. tRNA modification can thus change protein output by altering either the availability of functional tRNAs or the decoding efficiency of particular codons.

**Figure 1:**
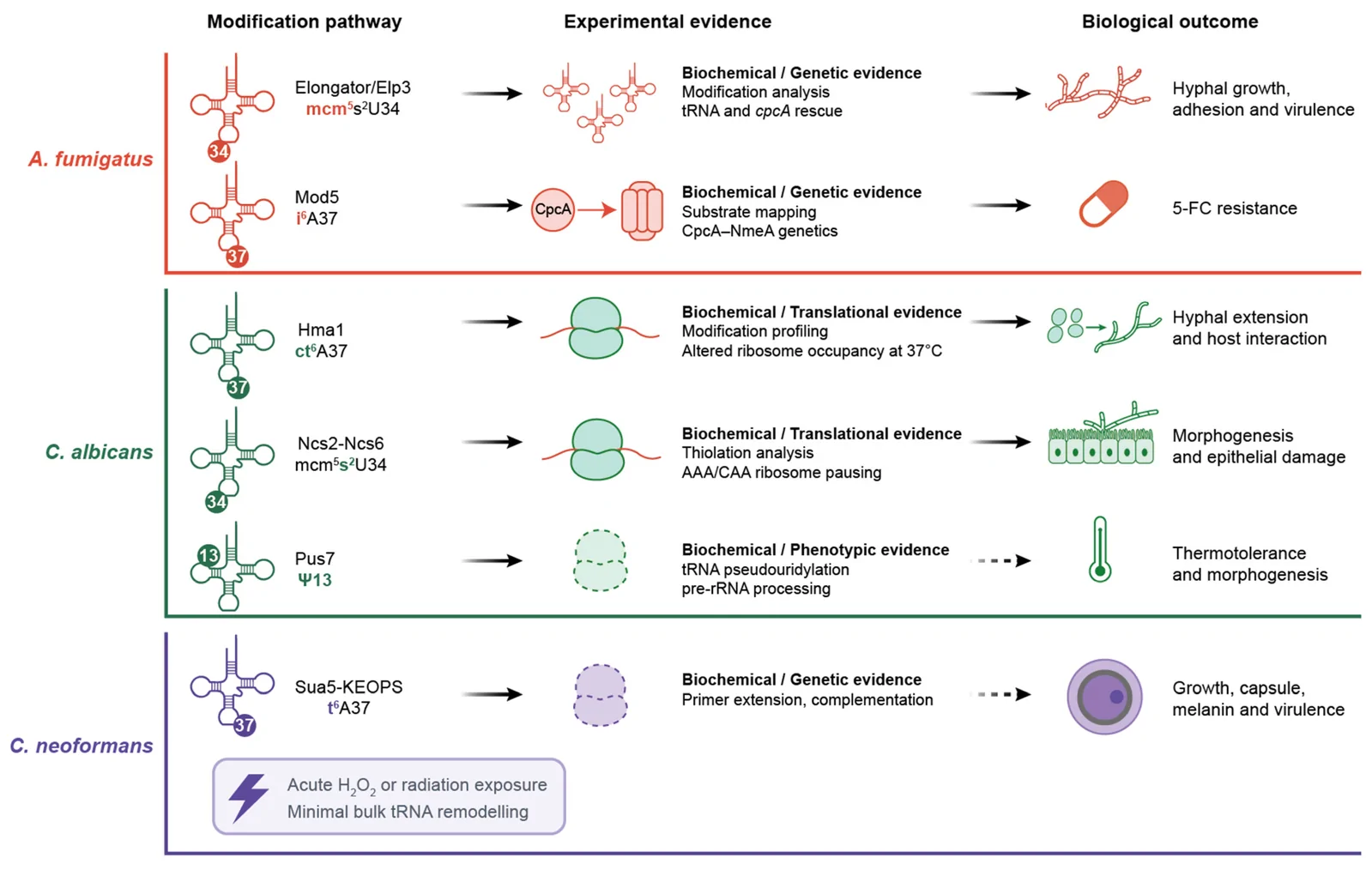
Evidence chains linking tRNA modification to infection-related phenotypes in human fungal pathogens. The principal modification pathways, mechanistic findings and biological outcomes reported in *A. fumigatus*, *C. albicans* and *C. neoformans* are summarized [18,19,20,21,22,23,24,25]. Solid connectors indicate experimentally supported links, whereas dashed connectors denote unresolved steps. Acute hydrogen peroxide or ionizing-radiation exposure caused little detectable remodeling of the bulk tRNA pool in *C. neoformans* [23]. 5-FC, 5-fluorocytosine.

Recent reviews have placed RNA modification within infectious-disease biology and have surveyed tRNA-modification pathways in plant-pathogenic fungi [10,16,17]. Studies of tRNA modification in human fungal pathogens are centered on *A. fumigatus*, *C. albicans* and *C. neoformans*. Across these species, conserved modification pathways have been linked to fungal development, stress adaptation, virulence and antifungal responses [18,19,20,21,22,23,24,25]. Direct ribosome-profiling evidence currently comes from *C. albicans*. Studies in *A. fumigatus* and *C. neoformans* combine biochemical characterization of modification reactions and substrates with genetic, proteomic and infection phenotypes, while the intervening changes in ribosome behavior remain less clearly defined. In this focused Review, we examine how far each pathway has been connected to substrate tRNAs, translation, protein output and infection-related phenotypes (Figure 1 and Table 1). We draw on evidence from yeast and plant-pathogenic fungi to clarify conserved biochemical mechanisms and illustrate causal tests that have not yet been applied in human fungal pathogens.

## 2. tRNA Modification and CpcA-Dependent Stress Responses in *Aspergillus fumigatus*

In *A*. *fumigatus*, disruption of two chemically distinct tRNA-modification pathways activates CpcA, the transcription factor that coordinates fungal cross-pathway control during amino-acid limitation. Elongator deficiency primarily impairs fungal development and virulence, whereas Mod5 deficiency alters antifungal susceptibility.

### 2.1. Elongator-Dependent Wobble Uridine Modification During Hyphal Development

Elongator initiates side-chain formation at the wobble uridine (U34) of cytosolic tRNA-Gln (UUG), tRNA-Glu (UUC) and tRNA-Lys (UUU), which decode CAA, GAA and AAA codons, respectively [13]. In *A. fumigatus*, deletion of any Elongator subunit impairs colony growth and conidiation. ELP3 is the catalytic subunit, and its deletion nearly abolishes the downstream product mcm^5^s^2^U in total tRNA [18]. Increasing the copy number of the three substrate tRNAs partly restores growth, galactosaminogalactan (GAG) production and adhesion, with tRNA-Gln (UUG) producing the strongest rescue. The incomplete rescue by any single tRNA indicates that several Elongator substrates or downstream responses contribute to the phenotype.

Reduced GAG production was considered a possible downstream cause of the *elp3* deletion phenotype. Restoring individual GAG-biosynthesis proteins improves adhesion and partly restores virulence, but not hyphal growth [18]. Proteomic analysis did not reveal a clear codon-biased protein signature, as proteins reduced in the *elp3* mutant were not strongly enriched in AAA, CAA or GAA codons. Instead, amino-acid biosynthetic enzymes increased and CpcA was activated. Deleting *cpcA* from the *elp3* background restores growth, conidiation, GAG production, adhesion and nearly full virulence [18]. CpcA activation is therefore a major contributor to the developmental and infection defects caused by Elongator loss.

### 2.2. Mod5-Mediated tRNA Modification in the 5-FC Response

Mod5 installs N^6^-isopentenyladenosine at position 37 (i^6^A37) in a restricted set of cytosolic and mitochondrial tRNAs. In *A. fumigatus*, deletion of *mod5* eliminates detectable i^6^A from total tRNA, and Nano-tRNAseq identifies cytosolic tRNA-Tyr, tRNA-Cys and tRNA-Ser families among its substrates [19]. Additional tRNA-Tyr improves growth during amino-acid limitation but does not restore 5-fluorocytosine (5-FC) sensitivity, separating the nutritional defect from the drug-response phenotype. Matched transcriptomic and proteomic analyses also do not identify a discrete group of codon-biased proteins that accounts for 5-FC resistance.

The resistance mechanism instead involves CpcA-dependent expression of the nucleobase exporter *nmeA* [19]. CpcA is activated before drug exposure in the *mod5* mutant and responds more strongly after 5-FC treatment. Increasing *nmeA* expression enhances resistance, whereas deleting *nmeA* largely restores drug sensitivity in the *mod5* background. Mod5-deficient cells also contain less 5-fluorouracil (5-FU) in total tRNA after treatment, consistent with lower intracellular availability of 5-FC-derived metabolites. Together, these data support a model in which 5-FC resistance is mediated by CpcA-dependent nucleobase export rather than a direct defect in the canonical 5-FC metabolic pathway.

Elongator and Mod5 deficiency place chemically distinct tRNA defects upstream of the same stress regulator. In the *elp3* mutant, CpcA accounts for much of the developmental and virulence phenotype, whereas in the *mod5* mutant, it acts through NmeA to reduce intracellular exposure to 5-FC-derived metabolites. Substrate-tRNA supplementation also separates the nutritional effects of Mod5 loss from its drug-response phenotype. The shared activation of CpcA reflects a common surveillance response, while the downstream outcome depends on the initiating tRNA defect and physiological condition [18,19].

## 3. tRNA Modification and Adaptation to Host Temperature in *Candida albicans*

Whereas the *A. fumigatus* studies link tRNA hypomodification mainly to cross-pathway control, work in *C. albicans* provides more direct evidence of tRNA-modification-dependent changes in ribosome behavior under host-temperature conditions.

### 3.1. Hma1-Mediated Translation at Host Temperature

Hma1 converts N^6^-threonylcarbamoyladenosine at position 37 (t^6^A37) to its cyclic derivative, ct^6^A37, in tRNAs with NNU anticodons. In *C. albicans*, deletion of *hma1* causes t^6^A to accumulate and eliminates ct^6^A, confirming the predicted modification reaction [20]. The mutant can initiate filamentation but forms shorter invasive hyphae and shows reduced expression of the hypha-associated gene *ece1*. It adheres to and invades oral epithelial cells less efficiently than the wild type and shows reduced virulence in an embryonated chicken egg model. Hma1 therefore contributes to hyphal extension and efficient epithelial adhesion and invasion but is not required for the initial yeast-to-hypha transition or for hyphal growth after invasion.

The translational phenotype is clearest at mammalian temperature. Hma1 loss produces a broad redistribution of ribosome occupancy at host temperature. Ribosome profiling detects little change in codon occupancy at 30 °C, whereas *hma1* deletion at 37 °C alters occupancy across several codon families decoded by ct^6^A-containing tRNAs [20]. Affected codons extend beyond those decoded by the direct tRNA substrates, indicating that loss of Hma1 has broader effects on elongation or translation initiation. Transcripts translated more efficiently in the wild type at 37 °C include regulators and effectors of filamentation and adhesion, such as *hgc1*, *brg1* and *als1*. Their temperature-associated changes in translation are consistent with the observed defects in hyphal extension and epithelial interaction.

### 3.2. Ncs2–Ncs6-Dependent U34 Thiolation and Codon-Specific Decoding

Unlike the broader changes in ribosome occupancy caused by *hma1* deletion, loss of Ncs2 produces a more codon-resolved translational defect. Ncs2 and Ncs6 thiolate mcm^5^U34 to form mcm^5^s^2^U34 in tRNA-Lys (UUU), tRNA-Gln (UUG) and tRNA-Glu (UUC) through the Uba4–Urm1 pathway [29,30]. In *C. albicans*, *ncs2* deletion removes thiolation from all three tRNAs, and ribosome profiling at 37 °C detects increased A-site occupancy at AAA and CAA codons [24]. Transcripts showing reduced ribosome footprint density are enriched in mitochondrial, membrane and hyphal functions, while the mutant forms fewer hyphae and causes less epithelial-cell damage. The direct modification assay and codon-specific occupancy changes provide the clearest evidence in *C. albicans* that impaired decoding accompanies defective morphogenesis and host interaction.

### 3.3. Pus7-Dependent Pseudouridylation and RNA Processing

Pus7 modifies tRNA and also contributes to pre-rRNA maturation in *C. albicans*. Primer-extension analysis identified Pus7-dependent pseudouridylation at U13 of tRNA-Glu (CUC), with U8 and U11 as additional candidate sites [25]. Loss of Pus7 causes accumulation of ITS1- and ITS2-containing pre-rRNA intermediates. The mutant also shows temperature-sensitive growth, defective filamentation, decreased cell-surface hydrophobicity and biofilm formation, with altered antifungal susceptibility and attenuated virulence in the Galleria mellonella infection model [25]. Pus7 therefore connects tRNA pseudouridylation and pre-rRNA maturation with thermotolerance and morphological plasticity, but the contribution of each RNA pathway has not been separated. Complementation with Pus7 variants that restore tRNA pseudouridylation without correcting pre-rRNA processing, or vice versa, could reveal which defect contributes to thermotolerance and morphogenesis.

Host temperature exposes several forms of RNA-dependent dysfunction in *C. albicans*. Hma1 loss redistributes ribosome occupancy across multiple codon families, whereas Ncs2 loss produces more localized pausing at AAA and CAA. Pus7 presents a different case in which tRNA pseudouridylation and pre-rRNA maturation change together. Similar defects in morphogenesis can therefore arise from codon-selective slowing, broader changes in ribosome behavior or disruption of another step in ribosome production.

## 4. tRNA Modification and Stress Adaptation in *Cryptococcus neoformans*

In *C. neoformans*, genetic evidence links cytosolic t^6^A synthesis to growth, stress adaptation and pathogenic fitness, whereas acute peroxide or radiation exposure produces little detectable change in bulk tRNA composition or modification abundance.

### 4.1. KEOPS and Sua5 Control of t^6^A Modification

Sua5 and KEOPS catalyze successive steps in t^6^A37 synthesis in ANN-decoding tRNAs. Sua5 produces threonylcarbamoyl-AMP, and KEOPS transfers the threonylcarbamoyl group to A37 [14,22]. *C. neoformans* contains the four conserved KEOPS subunits Pcc1, Kae1, Bud32 and Cgi121. Deletion of any KEOPS subunit gene alters the primer-extension profile of tRNA-Ile (AAU), whereas the non-ANN-decoding control tRNA-Val (AAC) is unaffected [21]. Reintroduction of BUD32 restores a wild-type-like tRNA-Ile profile, supporting a conserved role for cryptococcal KEOPS in t^6^A synthesis.

KEOPS mutants show defects in vegetative growth, sexual development, stress adaptation, capsule and melanin production, and pathogenic fitness. Loss of *cgi121* generally produces milder phenotypes than disruption of the other subunits, indicating unequal contributions within the complex [21]. The Sua5 study connects these pleiotropic KEOPS phenotypes more specifically to t^6^A synthesis. Deletion of *sua5* produces a similar tRNA defect and overlapping developmental and virulence-associated phenotypes [22]. A *sua5 bud32* double mutant is no more severely affected than either single mutant, supporting their action in the same pathway. Sua5 lacking its predicted mitochondrial targeting sequence remains functional, whereas deletion of the mitochondrial Kae1 paralogue QRI7 has little effect on growth or virulence-factor production. These results place the relevant activity in the cytosol and link the overlapping Sua5 and KEOPS phenotypes to t^6^A synthesis.

Disruption of KEOPS also changes transcriptional and proteomic programs associated with nutrient metabolism, sterol synthesis, iron transport and iron–sulfur cluster assembly [21,31]. These broad phenotypic responses show that loss of the pathway affects multiple physiological systems, but they do not distinguish primary translational defects from secondary consequences of impaired growth or stress adaptation. The combined KEOPS and Sua5 genetics therefore provide strong pathway-level evidence linking cytosolic t^6^A with cryptococcal pathogenic fitness. In contrast to the Hma1 and Ncs2 studies in *C. albicans*, however, direct effects on codon occupancy and protein production remain undefined.

### 4.2. Limited Remodeling of the tRNA Pool During Acute Oxidative Stress

The requirement for cytosolic t^6^A does not imply extensive remodeling of the tRNA pool during every stress response. In the environmental serotype D strain JEC21, hydrogen peroxide treatment for 1 h or exposure to ionizing radiation at 30 °C produced limited changes in both bulk tRNA composition and modified-nucleoside abundance [23]. The study defined significance as a twofold change with *p* < 0.05. By these criteria, LC–MS/MS showed that modified nucleosides including mcm^5^s^2^U and i^6^A were largely unchanged, while tRNA sequencing detected significant abundance changes in only 2 of 54 tRNAs after hydrogen peroxide treatment and 3 of 54 after irradiation [23]. Oxidative-response genes were induced under the same conditions, confirming that the cells had sensed and responded to the treatments. These measurements captured the bulk tRNA pool during acute stress at 30 °C and may have missed changes confined to individual tRNAs. The results therefore show that JEC21 did not undergo widespread tRNA remodeling under the tested conditions, but do not establish that the tRNA pool remains stable during infection.

## 5. Linking tRNA Modification to Translational Adaptation

### 5.1. From Modification Defects to Protein Output

Studies in human fungal pathogens connect tRNA modification to infection at different points in the evidence chain. In *C. albicans*, the Hma1 and Ncs2 studies combine direct modification measurements with ribosome profiling at 37 °C. Hma1 loss alters occupancy across several codon families, whereas Ncs2 loss increases A-site occupancy at AAA and CAA [20,24]. In *A. fumigatus*, substrate-tRNA supplementation and downstream genetics place Elongator and Mod5 defects upstream of CpcA-dependent responses, but the initiating changes in translation have not been identified [18,19]. Genetics in *C. neoformans* establishes a requirement for cytosolic t^6^A synthesis without directly measuring ribosome behavior or protein output [21,22]. No study in these pathogens has yet connected a defined modification and substrate tRNA to altered production of a specific infection-related protein. The evidence can be ordered from genetic and infection phenotypes, through identification of the modification and substrate tRNAs and direct measurement of translation or protein output, to functional rescue or recoding. The pathways and biological outcomes represented at each level are summarized in Table 1.

The missing decoding and protein-output links cannot be inferred simply from substrate identity or codon use. In *Saccharomyces cerevisiae*, U34 thiolation changes with nutrient availability and temperature [30,32,33,34]. Loss of U34 modifications causes ribosome pausing, activates starvation signaling and increases protein aggregation [35,36,37,38]. Ribosome pausing is not distributed evenly across all codons decoded by the affected tRNAs. Disome profiling detected severe collisions only at particular combinations of codons in the ribosomal P and A sites [39]. The effect of modification loss therefore depends on the physiological condition and the local sequence in which a sensitive codon occurs. Identifying an affected tRNA or counting its cognate codons is insufficient to predict which proteins will decline.

Studies in the plant pathogen *Magnaporthe oryzae* establish the downstream links more directly. Loss of Ncs2–Ncs6-dependent U34 thiolation increases ribosome occupancy at AAA, CAA and GAA and reduces infection-related proteins without corresponding transcript changes [26]. Target overexpression partly restores infection phenotypes, while synonymous recoding of the effector PWL2 restores its production in a thiolation-deficient strain [26,27]. Trm6–Trm61-dependent m^1^A58 affects a broader set of proteins. Loss of m^1^A58 weakens tRNA–eEF1 interactions and reduces translation of several ergosterol-biosynthesis proteins, leading to lower ergosterol abundance and impaired infection [28]. The U34 pathway demonstrates codon-dependent control of individual infection proteins, whereas m^1^A58 connects tRNA function to the coordinated output of a metabolic pathway. The plant-pathogen studies therefore suggest two mechanisms to examine in human fungal pathogens. Modification loss may disrupt the codon-dependent production of individual infection proteins or the coordinated translation of a functional pathway.

### 5.2. Cross-Species Comparison of Conserved tRNA-Modification Pathways

U34 pathways provide the clearest cross-species comparison because the available studies perturb different steps in related chemistry. Elongator initiates side-chain formation at U34, whereas Ncs2–Ncs6 supplies the subsequent thiolation step. Their disruption therefore does not generate identical hypomodified tRNA pools. Even so, impaired U34 modification repeatedly intersects nutrient homeostasis. Elongator loss activates CpcA in *A. fumigatus*, sulfur availability regulates U34 thiolation in *S. cerevisiae*, and U34 hypomodification in yeast activates starvation signaling even in nutrient-rich medium [18,30,32,35,36,37,38]. The *C. albicans* and *M. oryzae* studies extend this relationship to ribosome behavior because Ncs2–Ncs6 loss increases occupancy at codons decoded by the affected tRNAs [24,26,27]. Codon-specific slowing and nutrient signaling may therefore represent different levels of the response to inefficient U34-dependent decoding.

The downstream phenotype is also determined by which proteins encounter the decoding defect. Disome profiling shows that severe collisions occur at selected combinations of codons in the ribosomal P and A sites, rather than at every codon read by the affected tRNAs [39]. Transcript sensitivity will therefore depend on codon order, expression level, tRNA availability and the capacity to resolve stalled ribosomes. Fungi require different protein sets during hyphal development, epithelial interaction or plant invasion. The same tRNA defect can consequently engage nutrient surveillance in one setting but reduce production of individual infection proteins in another. Current studies also use different temperatures, nutrient sources, developmental stages and translational readouts, so not all apparent divergence can be attributed to species biology.

A37 pathways illustrate a second distinction. Hma1 loss in *C. albicans* prevents conversion of t^6^A to ct^6^A while leaving t^6^A present, whereas loss of Sua5 or KEOPS in *C. neoformans* interferes with t^6^A synthesis itself [20,21,22]. The resulting tRNA pools and potential decoding defects differ even though all of these pathways act at A37. Ribosome profiling after Hma1 loss captures a temperature-dependent translational phenotype, while the Sua5 and KEOPS studies establish t^6^A dependence through pathway genetics. Cross-species comparison therefore requires the chemical step, substrate tRNAs, physiological condition and mechanistic readout to be considered together. Conserved tRNA-modification pathways do not produce a fixed phenotype. Their output depends on both the molecular lesion and the proteins required under the condition examined.

## 6. Perspectives on tRNA Modification in Human Fungal Pathogenesis

### 6.1. Defining When Modification Dependence Emerges During Infection

Modification dependence may emerge only at a particular stage of infection. In *C. albicans*, loss of Hma1 or Ncs2 alters ribosome occupancy most clearly at mammalian temperature [20,24], whereas *C. neoformans* requires cytosolic t^6^A despite little bulk tRNA remodeling during acute peroxide or radiation exposure [21,22,23]. These observations separate a condition-specific requirement for a pathway from a detectable change in modification abundance.

The relevant transition is likely to differ among pathogens. In *A. fumigatus*, dependence may emerge as conidia germinate and establish invasive hyphae. In *C. albicans*, it may arise during hyphal induction or epithelial invasion, whereas in *C. neoformans*, it may accompany capsule induction or growth after macrophage uptake [6,7,8]. Following wild-type and modification-deficient strains through these stages would show when each pathway begins to influence fungal development or survival. Measuring modification abundance in parallel would reveal whether the transition remodels the tRNA pool or increases demand for modifications that are already present. Using comparable fungal biomass and developmental stages would prevent differences in growth rate from obscuring the timing of the response.

### 6.2. Measuring tRNA Modification During Infection

Most measurements of fungal tRNA modification have been made in culture, leaving the tRNA state during infection largely unknown. Analysis of infected tissue must distinguish changes in modification stoichiometry from changes in tRNA abundance, aminoacylation or fungal biomass. This is difficult because pathogen tRNAs are scarce relative to host RNA and modified nucleosides can bias reverse transcription [40]. Bulk measurements from infected organs introduce a further problem because they average fungal populations in different physiological states. A response confined to germinating conidia, invasive hyphae or intracellular cells may be diluted by fungi at other stages of infection. Sampling defined tissues and infection stages, or enriching particular fungal populations where possible, would preserve this biological resolution. Modification abundance should also be interpreted relative to the abundance of the corresponding tRNA species. An apparent change in a modified nucleoside may reflect altered modification stoichiometry, a change in the amount of the tRNA carrying it, or both. Measuring these variables in the same samples would distinguish widespread remodeling of the fungal tRNA pool from a localized response obscured in whole-organ measurements.

Once the relevant tissue or infection stage has been selected, fungal-specific enrichment can reduce the host background before tRNA analysis. Sequencing methods that overcome reverse-transcription barriers can improve estimates of tRNA abundance and modification-dependent signatures [41,42,43]. Nanopore methods retain modification-associated signals [44,45], and a newer nanopore approach can distinguish charged from uncharged tRNAs [46]. PRAISE provides quantitative, site-specific pseudouridine profiling in small RNAs and may help define Pus7 substrates [47]. LC–MS/MS remains necessary to confirm modification identity and abundance. Applying these measurements to the same infection samples would reveal whether host conditions remodel the fungal tRNA pool or increase dependence on modifications that are already present.

### 6.3. Linking tRNA Modification to Antifungal Treatment

Current evidence does not establish tRNA-modification enzymes as selective antifungal targets. The pathways discussed here are generally conserved across eukaryotes, and their disruption often causes broad growth defects. A more tractable question is whether modification state alters the activity of an established antifungal under a defined physiological condition. In *A. fumigatus*, Mod5 deficiency increases 5-fluorocytosine resistance through CpcA-dependent *nmeA* expression and nucleobase export [19]. In *M. oryzae,* impaired m^1^A58 methylation increases the effect of interfering with ergosterol biosynthesis [28]. Reduced tRNA modification can therefore either promote resistance or increase sensitivity to disruption of another pathway, depending on the modification and drug involved. The m^1^A58 result suggests that a modification defect can expose a vulnerability in sterol metabolism, whereas the Mod5 study shows how compensatory signaling and drug export can produce resistance. Both observations come from experimentally modified strains, and their prevalence among clinical isolates remains to be determined. Direct inhibition of the conserved catalytic enzymes would also face problems of fungal selectivity. Fungal-specific regulatory connections downstream of these pathways, including the CpcA–NmeA response, may provide more selective points of intervention. Testing these connections with established antifungals under host-associated conditions and across genetically diverse isolates could reveal when tRNA modification contributes to drug susceptibility or treatment failure.

## 7. Conclusions

tRNA modification is emerging as a layer of translational regulation during fungal adaptation to the host. Its contribution does not necessarily require a large change in modification abundance. A relatively stable modification can become limiting when host temperature, nutrient availability, morphological transitions or drug exposure alter the demand for particular tRNAs and proteins. Current studies connect modification defects with codon decoding, ribosome behavior, stress signaling and infection-related phenotypes, indicating that different pathways act at distinct points in translation and cellular adaptation. Establishing which modified tRNAs become limiting and which proteins are affected during infection will clarify how tRNA modification contributes to fungal pathogenicity and antifungal response.

## Figures and Tables

**Table 1:** Evidence linking tRNA modification to fungal pathogenicity, stress adaptation and translational control.

Fungal System	Enzyme or Pathway	Modification and Principal Substrates	Biological Outcome	Evidence Type and Key Boundary	Reference
*A. fumigatus*	Elongator/Elp3	Elongator-dependent U34 side-chain pathway in tRNA-Gln(UUG), tRNA-Glu(UUC) and tRNA-Lys(UUU); pathway disruption reduces mcm^5^s^2^U34	Hyphal growth, conidiation, galactosaminogalactan production, adhesion and virulence	Biochemical and genetic evidence; ribosome-level effects not examined	[18]
*A. fumigatus*	Mod5	i^6^A37 in cytosolic tRNA-Tyr, tRNA-Cys and tRNA-Ser, with additional mitochondrial substrates	5-fluorocytosine response, CpcA activation and NmeA-dependent nucleobase export	Biochemical and genetic evidence; the translational trigger of CpcA activation is unknown	[19]
*C. albicans*	Hma1	Conversion of t^6^A37 to ct^6^A37 in t^6^A-containing tRNAs	Filamentation at host temperature, epithelial interaction and virulence	Biochemical and ribosome-profiling evidence	[20]
*C. albicans*	Ncs2–Ncs6	mcm^5^s^2^U34 in tRNA-Lys(UUU), tRNA-Gln(UUG) and tRNA-Glu(UUC)	Morphogenesis, epithelial interaction and host-cell damage	Biochemical and codon-resolved ribosome-profiling evidence	[24]
*C. albicans*	Pus7	Ψ13 in tRNA-Glu(CUC), with U8 and U11 as candidate sites	Thermotolerance, filamentation, biofilm formation, drug response and virulence	Biochemical and phenotypic evidence; tRNA and pre-rRNA pathways are both affected	[25]
*C. neoformans*	Sua5–KEOPS pathway	Cytosolic t^6^A37 in ANN-decoding tRNAs	Growth, sexual development, stress adaptation, capsule and melanin production, and pathogenic fitness	Biochemical and genetic evidence; ribosome-level effects not examined	[21,22]
*M. oryzae*	Ncs2–Ncs6 and Uba4–Urm1	mcm^5^s^2^U34 in tRNA-Lys(UUU), tRNA-Gln(UUG) and tRNA-Glu(UUC)	Appressorium formation, production of infection-related proteins and plant virulence	Biochemical, ribosome-profiling and causal-rescue evidence	[26,27]
*M. oryzae*	Trm6–Trm61	m^1^A58 in 51 tRNAs	Ergosterol biosynthesis and plant infection	Biochemical and translational evidence	[28]
Clinical *S. cerevisiae* isolate	Natural Ncs2 variant	Increased mcm^5^s^2^U34 formation	Thermotolerance, protein homeostasis and virulence	Genetic, biochemical and infection evidence	[24]

Note: *M. oryzae* and the clinical *S. cerevisiae* isolate are included as comparative systems that provide mechanistic context, not as direct evidence from the three human fungal pathogens reviewed here.

## Data Availability

No new data were created or analyzed in this study. Data sharing is not applicable to this article.
